# Characterization of Kinesin Family Member 2C as a Proto-Oncogene in Cervical Cancer

**DOI:** 10.3389/fphar.2021.785981

**Published:** 2022-01-27

**Authors:** Jing Yang, Zimeng Wu, Li Yang, Ji-Hak Jeong, Yuanhang Zhu, Jie Lu, Baojin Wang, Nannan Wang, Yan Wang, Ke Shen, Ruiqing Li

**Affiliations:** ^1^ Department of Obstetrics and Gynecology, The Third Affiliated Hospital of Zhengzhou University, Zhengzhou, China; ^2^ Henan International Joint Laboratory of Ovarian Malignancies, Zhengzhou, China; ^3^ Zhengzhou Key Laboratory of Endometrial Disease Prevention and Treatment, Zhengzhou, China; ^4^ Research Institute of Pharmaceutical Sciences, College of Pharmacy, Kyungpook National University, Daegu, South Korea

**Keywords:** KIF2C, cervical cancer, P53 signaling pathway, molecularly targeted therapy, pan-cancer analysis

## Abstract

Kinesin family member 2C (*KIF2C*) is known as an oncogenic gene to regulate tumor progression and metastasis. However, its pan-cancer analysis has not been reported. In this study, we comprehensively analyzed the characteristics of *KIF2C* in various cancers. We found that *KIF2C* was highly expressed and corresponded to a poor prognosis in various cancers. We also found a significant correlation between KIF2C and clinicopathological characteristics, particularly in cervical cancer, which is the most common gynecological malignancy and is the second leading cause of cancer-related deaths among women worldwide. *KIF2C* mutation is strongly associated with the survival rate of cervical cancer, and *KIF2C* expression was significantly upregulated in cervical cancer tissues and cervical cancer cells. Moreover, *KIF2C* promoted cervical cancer cells proliferation, invasion, and migration *in vitro* and as well increased tumor growth *in vivo*. *KIF2C* knockdown promotes the activation of the p53 signaling pathway by regulating the expression of related proteins. The rescue assay with KIF2C and p53 double knockdown partially reversed the inhibitory influence of KIF2C silencing on cervical cancer processes. In summary, our study provided a relatively comprehensive description of *KIF2C* as an oncogenic gene and suggested *KIF2C* as a therapeutic target for cervical cancer.

## Introduction

Kinesin family member 2C (*KIF2C*), also known as mitotic centromere associated kinesin (*MCAK*), is the most representative member of the kinesin family-13 (Kinesin-13) (Ritter et al., 2015). It participates in the functions of microtubules and regulates mitosis and cell cycle, involving in the disaggregation and formation of secondary spindles and the separation of chromosomes (DeLuca et al., 2006; Ganguly et al., 2008; Hedrick et al., 2008; Huang et al., 2009). Because *KIF2C* is involved in the regulation of tumor development, proliferation, and metastasis, it has been considered as a candidate promoting factor for breast cancer, liver cancer, lung cancer, bladder cancer, colon cancer, and other cancers (Bai et al., 2019; Wei et al., 2020; Yang et al., 2020). Shi et al. reported that *KIF2C* is activated by the Wnt/β-catenin signaling pathway and plays a key role in mTORC1 signal transduction and hepatocellular carcinoma progression through its interaction with *TbC1D7* (Wei et al., 2020). Gan et al. (2019) revealed that *KIF2C* has a carcinogenic effect in non-small cell lung cancer and is negatively regulated by miR-325-3p. Yang et al. (2020) showed that *circRGNEF* promotes the occurrence and development of bladder cancer by directly targeting miR-548 and upregulating the expression of *KIF2C*.

Cervical cancer is one of the most common gynecological malignancies with high morbidity and mortality, and it is the second leading cause of cancer-related deaths among all female malignant tumors (Siegel et al., 2021). The most common pathological type of cervical cancer is cervical squamous cell carcinoma (Zhao et al., 2019). When cervical cancer patients are diagnosed and treated in the early tumor stage, the chance of survival can be increased and may push the 5-year survival rate to reach as high as 90% (Das, 2021). However, the early symptoms of cervical cancer are not obvious, and 70% of cervical cancer patients are in the middle and late stages of the disease when they are diagnosed with cancer before receiving treatment. Additionally, approximately 31% of cervical cancers relapse after the treatment, and most of the recurrence occurs within 2 years after the initial diagnosis (Zhao and Qiao, 2019; Brisson et al., 2020). The treatment of recurrent cervical cancer is very difficult, and its prognosis is very poor, causing the 1-year survival rate to be only 8–12% (Cohen et al., 2019). Therefore, early treatment provides life-saving, and more and more attention has been paid to finding meaningful therapy targets for cervical cancer.

In the present study, we used The Cancer Genome Atlas (TCGA) project and Gene Expression Omnibus databases to conduct a pan-cancer analysis of *KIF2C* for the first time, including gene expression, survival status, and genetic alteration. Interestingly, we identified *KIF2C* that has a significant value for the research in cervical cancer. Hence, we experimentally confirm that KIF2C expression was increased in cervical cancer tissues and cervical cancer cells. Through *in vivo* experiments, *KIF2C* was found to play an important role in the development of cervical cancer. In addition, RNA sequencing analysis of cervical cancer cells with *KIF2C* knockdown showed that differentially expressed genes were enriched in the p53 signaling pathway. Further analysis of the expression of proteins in the p53 signaling pathway showed that the knockdown of *KIF2C* could induce the activation of the p53 signaling pathway. Beyond this, rescue experiments demonstrated that knockdown of p53 partially reversed the inhibitory influence of knockdown KIF2C silencing on cervical cancer processes. Our findings provide new insights into the functional and mechanistic link between the *KIF2C* and P53 signaling pathways in cervical cancer, which might help discover adjuvant therapy for patients with cervical cancer.

## Materials and Methods

### Gene Expression Analysis

The KIF2C expression of tumor and adjacent normal tissues profile was analyzed using the TIMER 2.0 database (https://cistrome.shinyapps.io/timer/), a bioinformatics tool for analyzing and visualizing TCGA (Li et al., 2020a). The 33 TCGA cancer types analyzed are shown in Table 1. The gene expression levels are represented as log2 transcript per million (TPM) values. For some tumor tissues without normal tissues, normal expression comparison analyses, the TPM of the given genes from TCGA, and matched GETx (Genotype-Tissue Expression) normal tissue RNA sequencing (RNA-seq) datasets were visualized by GEPIA2 (http://gepia2.cancer-pku.cn/#analysis) (Tang et al., 2017). The cutoff for significance was chosen at *p* = 0.01. The tumor pathological staging map was also obtained from GEPIA2. TPM values were calculated, and the expression levels of genes were presented using the Log2 (TPM + 1) scale.

**TABLE 1:** TCGA cancer types analyzed.

Cancer type	Full name	Number
ACC	Adrenocortical carcinoma	79
BLCA	Bladder urothelial carcinoma	408
BRCA	Breast invasive carcinoma	1,100
CESC	Cervical squamous-cell carcinoma and endocervical adeno-carcinoma	306
CHOL	Cholangiocarcinoma	36
COAD	Colon adenocarcinoma	287
DLBC	Lymphoid neoplasm diffuse large B-cell lymphoma	48
ESCA	Esophageal carcinoma	185
GBM	Glioblastoma multiforme	166
HNSC	Head and neck squamous cell carcinoma	522
KICH	Kidney chromophobe	66
KIRC	Kidney renal clear cell carcinoma	534
KIRP	Kidney renal papillary cell carcinoma	291
LAML	Acute myeloid leukemia	173
LGG	Brain lower-grade glioma	530
LIHC	Liver hepatocellular carcinoma	373
LUAD	Lung adenocarcinoma	517
LUSC	Lung squamous cell carcinoma	501
MESO	Mesothelioma	87
OV	Ovarian serous cystadenocarcinoma	307
PAAD	Pancreatic adeno-carcinoma	179
PCPG	Pheochromocytoma and paraganglioma	184
PRAD	Prostate adenocarcinoma	498
READ	Rectum adenocarcinoma	95
SARC	Sarcoma	263
SKCM	Skin cutaneous melanoma	472
STAD	Stomach adenocarcinoma	415
TGCT	Testicular germ-cell tumors	156
THCA	Thyroid carcinoma	509
THYM	Thymoma	120
UCEC	Uterine corpus endometrial carcinoma	370
UCS	Uterine carcinosarcoma	57
UVM	Uveal melanoma	80

Protein expression of KIF2C was evaluated using the UALCAN online analysis software (http://ualcan.path.uab.edu/index.html), which is a comprehensive web resource for analyzing cancer-omics data including Clinical Proteomic Tumor Analysis Consortium analysis in various tumor types (Chen et al., 2019).

### Survival Analysis

The correlation between KIF2C messenger RNA (mRNA) expression and overall survival (OS) and disease-free survival (DFS) was predicted using the PrognoScan database (http://www.abren.net/PrognoScan/) and GEPIA2. PrognoScan database is also a collection of publicly available cancer microarray datasets and a tool for assessing the biological relationships between gene expression and prognosis, providing a convenient platform to evaluate potential tumor markers and therapeutic targets (Mizuno et al., 2009). The threshold was set as a Cox *p*-value < 0.05.

### Genetic Alteration Analysis

Genomic alteration data were analyzed through the cBio Cancer Genomics Portal (http://cbioportal.org) (Cerami et al., 2012; Gao et al., 2013), which collects multidimensional cancer genomics dataset. We observed the alteration frequency and mutation type of all 33 tumors. We also obtain the data on the overall disease-free, progression-free, and DFS differences for TCGA cancer cases with or without *KIF2C* genetic alteration.

### The Human Protein Atlas

HPA provides tissue and cell distribution information of 26,000 human proteins (Uhlén et al., 2015). In this database, researchers used highly specific antibodies and immunoassay techniques to detect the expression of each protein in 64 cell lines, 48 human normal tissues, and 20 tumor tissues in detail. In this study, we conducted HPA to observe the localization of KIF2C in cells and compare the protein expression of KIF2C between normal tissue and carcinoma tissue.

### LinkedOmics Database

The LinkedOmics database 8 (http://linkedomics.org) (Vasaikar et al., 2018) is an online open-access powerful bioinformatics platform, which includes multi-omics information and clinical data involving 11,158 patients and 32 cancer types in TCGA project. We used the Linkedomics platform to analyze the relationship between the expression level of KIF2C in TCGA database and the clinical–pathological characteristics of patients.

### Tissue Collection and Immunohistochemistry

Tissue specimens were obtained from patients who underwent hysterectomy and pathological diagnosis in the Department of Pathology of The Third Affiliated Hospital of Zhengzhou University (Zhengzhou, China) from 2017 to 2021. Paraffin-embedded histological specimens of 35 cervical squamous tissue samples, 25 cervical adenocarcinoma tissue samples, and 40 normal cervical tissues samples were sectioned (thickness 5 μm). All the patients had not received any antitumor therapy before their diagnoses. The project was approved by the Medical Ethics Committee of the Third Affiliated Hospital of Zhengzhou University (ethics approval number: 2021-080-01) and performed in accordance with the principles of the Declaration of Helsinki.

Immunohistochemistry and scoring criteria were performed as described (Soumaoro et al., 2004). Briefly, the tissue sections were deparaffinized in xylene, rehydrated in graded ethanol, and soaked in distilled water. After heat-mediated citric acid antigen retrieval, the tissue sections were incubated with the primary antibody for KIF2C (DF7348, rabbit antihuman, 1:100, Affinity) and Ki-67 (ab92742, rabbit antihuman, 1:500, Abcam) at 4°C overnight and incubated with horseradish peroxidase-conjugated antibody for 60 min at room temperature. Staining was developed by incubating with diaminobenzidine. Finally, these processed tissue sections were observed under a microscope and then counterstained with hematoxylin. The results were scored independently by two pathologists. A total score of >3 points was considered a high expression, and ≤3 points was considered low expression.

### Cell Culture

Cervical cancer cell lines SiHa, HeLa, C33a, and Caski and normal cervical cell line HCerEpic were borrowed from the relevant research group with our laboratory. All cells were cultured in Dulbecco’s modified Eagle’s medium (Solarbio, SuoLaibao Technology Co., Ltd.) supplemented with 10% fetal bovine serum (Gibco, Thermo Fisher Scientific, Inc.) and incubated at 37°C in a moist atmosphere containing 5% CO_2_.

### Knockdown of KIF2C by Lentivirus Transfection

When C33a and SiHa cells reached 20–30% confluence on the day of transfection, two lentiviral stocks (named GV248-shKIF2C and GV248-shCon, purchased from Genechem, Shanghai, China) were transduced into the cells with HitransG A, respectively. Stable cell lines were screened with 0.25 μg/ml puromycin. The cells were collected when 80–90% of the cells with green fluorescence were grown under a fluorescence microscope, then quantitative real-time polymerase chain reaction (qRT-PCR) and Western blot analysis were used to analyze the level of *KIF2C* expression.

### Knockout of p53 by CRISPR-Cas9

CRISPR/Cas9 p53 knockdown and its control plasmids were kindly provided by Dr. Qi Zhang; then, the plasmids were transfected into SiHa-sh-KIF2C and SiHa-sh-NC cervical cancer cells, respectively. The cells were transfected using JetPrime transfection reagent (Polyplus transfection, Illkirch, France) according to the manufacturer’s protocol; then, Western blot analysis was used to analyze the level of p53 expression.

### Overexpression of KIF2C by Plasmid

For overexpression, the plasmid (vector name: GV657) for human KIF2C and negative control were synthesized by Genechem (Shanghai, China), then transfected into Caski cell using JetPrime transfection reagent (Polyplus transfection, Illkirch, France) according to the manufacturer’s protocol using 1=µl of JetPrime for 1 µg of plasmid DNA. After being transfected with 24 h, the protein was collected, and Western blotting was used to verify the transfection efficiency.

### Quantitative Real-Time Polymerase Chain Reaction Analysis

According to the supplier’s instructions, synthesis of complementary DNA by extracting 1 μg total RNA with Trizol (Cwbio, Beijing, China) and SYBR reverse transcription reagent (US Everbright Inc., Suzhou, China). The ratio of target glyceraldehyde 3-phosphate dehydrogenase expressed the relative expression of target gene mRNA. The sequences for the primers are as follows:

GADPH, Forword, 5′-TGA​CTT​CAA​CAG​CGA​CAC​CCA-3′;
Reverse, 5′-CAC​CCT​GTT​GCT​GTA​GCC​AAA-3′;
KIF2C, Forword, 5′-GGA​GGA​GAA​GGC​TAT​GGA​AGA-3′;
Reverse, 5′-TCG​CAG​GGC​TGA​GAA​ATG-3′.

The relative gene expression was normalized to control by the 2^−ΔΔCT^ method.

### Western Blot Analysis

Total proteins from cells were extracted with radioimmunoprecipitation assay lysis buffer (Solarbio, SuoLaibao Technology Co., Ltd.). Western blot analysis was carried out by first electrophoresing the proteins through a sodium dodecyl sulfate–polyacrylamide gel electrophoresis gel and subsequently transferring to 0.45-µm polyvinylidene fluoride membrane (Millipore, Ireland). Immunoblotting was performed using antibodies against indicated proteins: *KIF2C* (DF7348, rabbit antihuman, 1:1,000, Affinity), *p21* (10355-1-AP, rabbit antihuman, 1:1,000, Proteintech), *p53* (sc126, mouse antihuman, 1:, Santa), *Bax* (50599-2-Ig, rabbit antihuman, 1:5,000, Proteintech), *bcl-2* (60178-1-Ig, mouse antihuman, 1:2,000, Proteintech), and GADPH (bs2188R, rabbit antihuman, 1:5,000, Bioss). Glyceraldehyde 3-phosphate dehydrogenase was used as an endogenous control. The transferred membranes were blocked with 5% milk for 2 h and then incubated with the indicated primary antibody overnight at 4°C. Then, the membranes were incubated with the appropriate secondary antibodies (1:9,000) at room temperature for 1 h. Finally, the electrogenerated chemiluminescence solution (A:B = 1:1) was added onto the membrane, and the front of the membrane was placed on the glass plate. The GelView 6000Plus was used to process the pictures after exposure.

### Cell Proliferation Assay

The successfully transfected cervical cancer cells (C33a, SiHa, and Caski) were inoculated in 96-well plates with 4 × 10^3^ cells per well. A 10-µl CCK-8 solution (US Everbright Inc., Suzhou, China) was added to each well after plating for 12, 24, 48, 72, and 96 h. When the cells were further incubated for 3 h, a microboard reader (Thermo Scientific) was used to measure the spectrometric absorbance at the wavelengths of 450 nm.

### Colony Formation Assay

After transfection, the cells were inoculated in six-well plates with 600 per well and cultured 2 weeks. Colonies were fixed with methanol for 20 min and subsequently stained with 0.1% crystal violet for 30 min. Colonies with more than 60 cells were defined as positive.

### Migration and Invasion Assay

For this assay, serum-free C33a, SiHa, and Caski cells were seeded to the upper chamber with 5 × 10^4^ cells in 100 µl, and the medium containing 20% fetal bovine serum was used as a chemoattractant in the lower chamber. After incubating for 36 h, migrated cells were fixed by formaldehyde and stained with crystal violet. For invasion assay, cells were also suspended in serum-free medium and counted and seeded in the upper chamber at a concentration of 5 × 104 cells in 100 µl; the only difference was spreading the Matrigel Basement Membrane Matrix (BD Bioscience, United States) on the upper chamber before adding the cells. The number of migrated and invaded cells was captured and calculated using a microscope.

### RNA-Sequencing and Data Analysis

Total RNAs were extracted from successfully transfected cervical cancer cells using Trizol reagent (Cwbio, Beijing, China). After a primary test for RNA quality and integrity, the sequencing samples were finally sequenced on Novaseq 6000 sequencer (Illumina) with PE150 model. The raw sequencing data were mapped to the human genome using STRA 2.5 software. Reads mapped to the exon regions of each gene were counted by featureCounts, and then reads per kilobase of transcript per million reads mapped was calculated. Differentially expressed (*p*-value cutoff of 0.05; the fold-change cutoff of 2) genes between groups were identified using the edgeR package. For further precise analysis, Gene Ontology (GO) analysis and Kyoto Encyclopedia of Genes and Genomes (KEGG) enrichment analysis for differentially expressed genes were conducted by KOBAS 2.1 software (*p*-value < 0.05; false discovery rate < 0.05). The RNA sequencing analysis and data analysis were performed with the help of Seqhealth cooperation (Wuhan, China). RNA-seq data have been deposited to National Center for Biotechnology Information Sequence Read Archive (Sequence Read Archive accession: PRJNA767113).

### *In Vivo* Experiments

Four- to 5-week-old female BALB/c nude mice (SPF Biotechnology Co., Ltd., Beijing, China) were used in this study. The control and experimental cervical cancer cells (2 × 10^6^) were transplanted to establish the subcutaneous xenograft model. Tumor volumes were measured every 7 days. After 4 weeks, the mice were killed, and tumor weight was examined. The tumor volumes were calculated by the formula: tumor volume = length × width^2^/2. Animal research was approved by the Animal Care and Use Committee of Zhengzhou University and was conducted in accordance with the Animal Care Guidelines of Zhengzhou University.

### Statistical Analysis

Statistical analysis of all data was done using SPSS 26.0 software. The measurement data with normal distribution was exhibited as the mean ± standard deviation. The statistical significance between the two groups was compared with the two-tailed Student’s t-test. Pearson’s chi-square test analyzed the association between KIF2C expression and clinicopathological features. *p* < 0.05 was considered statistically significant. All cell experiments had three repetitions.

## Results

### Database Analysis for KIF2C Expression in Various Cancers

**FIGURE 1:**
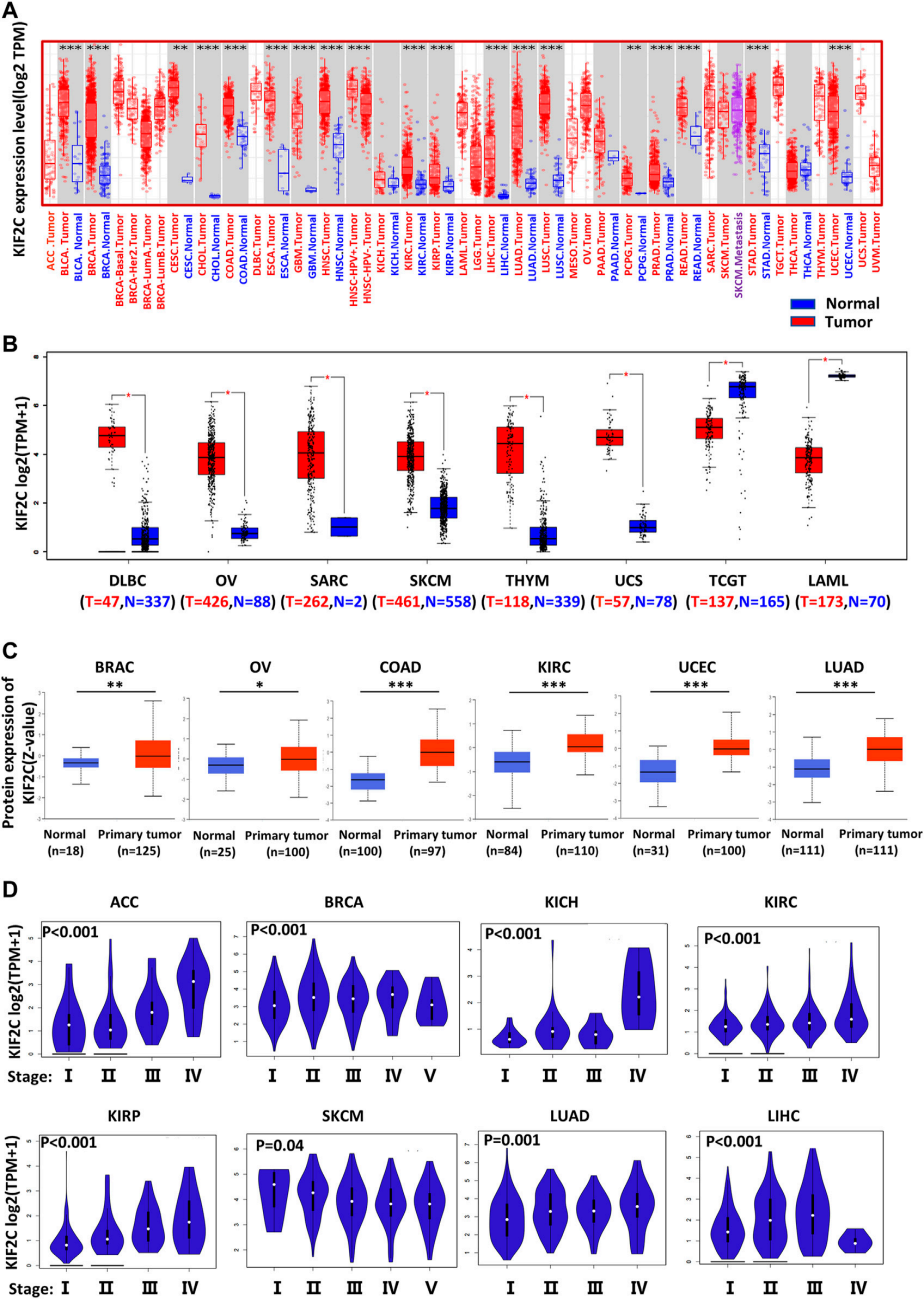
Different expression of *KIF2C* in various tumors and pathological stages. **(A)** *KIF2C* expression in 33 tumor types and paracancer tissues determined by TIMER. **(B)** Tumor types of DLBC, OV, SARC, SKCM, THYM, UCS, TGCT, and LAML were analyzed by box plot, for which corresponding normal tissues of GTEx database were included as controls. **(C)** Diagrams of quantified proteins analyzed by Clinical Proteomic Tumor Analysis Consortium in BRAC, OV, COAD, KIRC, UCEC, and LUAD. **(D)** Expression of *KIF2C* in different tumor stages. Log2 (TPM+1) was applied for log-scale. *p < 0.05; **p < 0.01; ***p < 0.001.

In the TIMER database, we investigated the expression of KIF2C in pan-cancer. The GEPIA2 database supplemented part of cancers without normal samples. The results are shown in Figures 1A,B. As expected, KIF2C expression was significantly increased in most tumor tissues compared with normal tissues, including TCGT and LAML. However, there was no significant difference between tumor and normal tissues in ACC and LGG. As there were no normal samples studied in either dataset, an analysis could not be performed for MESO and UVM. In addition, the protein expression level of KIF2C was also shown in a similar manner. Clinical Proteomic Tumor Analysis Consortium analysis showed that the protein expression of KIF2C was higher in primary tissues of BRAC, OV, COAD, KIRC, UCEC, and LUAD compared with those in normal tissues (Figure 1C). All the results were statistically significant (*p < 0.05*). Next, we used the UALCAN database to analyze the correlation between *KIF2C* mRNA expression and clinicopathological stages. Expression of *KIF2C* was highly associated with advanced clinical stage (Figure 1D) of ACC (*p* < 0.001), BRCA (*p* < 0.001), KICH (*p* < 0.001), KIRC (*p* < 0.001), KIRP (*p* < 0.001), SKCM (*p* = 0.04), LUAD (*p* = 0.001), and LIHC (*p* < 0.001) but not others.

### Survival Analysis for KIF2C in Various Cancers

**FIGURE 2:**
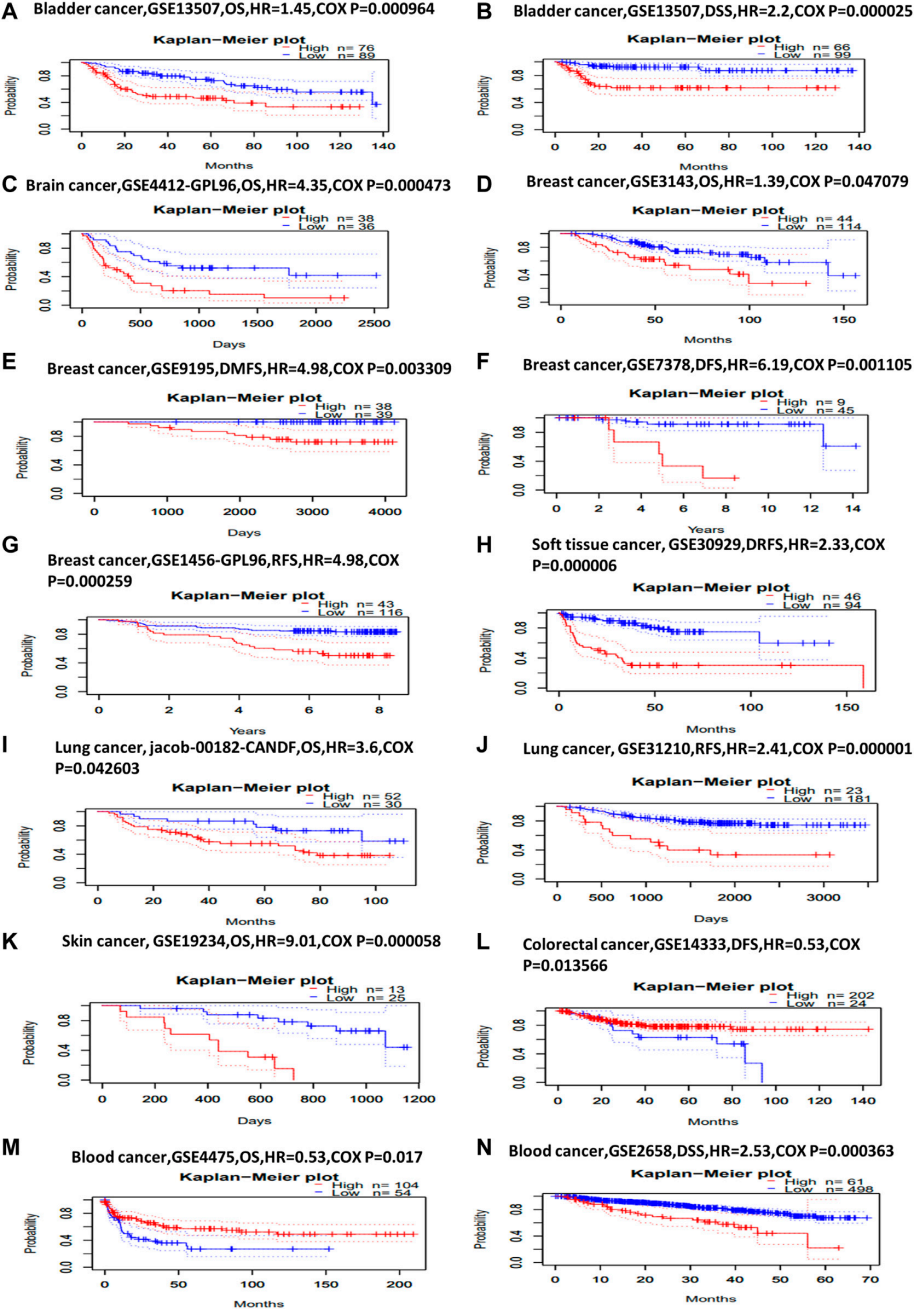
Kaplan–Meier survival curves comparing high and low expression of *KIF2C* in different cancer types in PrognoScan. **(A)** OS (*n* = 165) in bladder cancer cohort GSE13507. **(B)** DSS (*n* = 165) in bladder cancer cohort GSE13507. **(C)** OS (*n* = 74) in brain cancer cohort GSE4412-GPL96. **(D)** OS (*n* = 158) in breast cancer cohort GSE3143. **(E)** Distant metastasis-free survival (*n* = 77) in breast cancer cohort GSE9195. **(F)** DFS (*n* = 54) in breast cancer cohort GSE7378. **(G)** RFS (*n* = 159) in breast cancer cohort GSE1456-GPL96. **(H)** Distant relapse-free survival (*n* = 140) in soft-tissue cancer cohort GSE30929. **(I)** OS (*n* = 82) in lung cancer cohort jacob-00182-CANDF. **(J)** RFS (*n* = 204) in lung cancer cohort GSE31210. **(K)** OS (*n* = 38) in skin cancer cohort GSE19234. **(L)** DFS (*n* = 226) in colorectal cancer cohort GSE14333. **(M)** OS (*n* = 158) in blood cancer cohort GSE4475. **(N)** DSS (*n* = 559) in blood cancer cohort GSE2658.

Next, we investigated *KIF2C* prognostic value using PrognoScan and GEPIA2 database. In PrognoScan, high *KIF2C* expression is associated with worse OS [hazard ratio (HR) = 1.45, Cox *p* = 0.000964] and disease-specific survival (DSS, HR = 2.2, Cox *p* = 0.000025) in bladder cancer (Figures 2A,B), with worse OS (HR = 4.35, Cox *p* = 0.000473) in brain cancer (Figure 2C), with worse OS (HR = 1.39, COX *p* = 0.047079), distant metastasis-free survival (HR = 4.98, COX *p* = 0.003309), DFS (HR = 6.19, COX *p* = 0.001105), and recurrence-free survival (RFS, HR = 4.98, COX *p* = 0.000259) in breast cancer (Figures 2D–G), with worse distant relapse-free survival (HR = 2.33, COX *p* = 0.000006) in soft tissue cancer (Figure 2H), with worse OS (HR = 3.6, COX *p* = 0.042603), RFS (HR = 2.41, COX *p* = 0.000001) in lung cancer (Figures 2I,J) and with worse OS (HR = 9.01, COX *p* = 0.000058) in skin cancer (Figure 2K). Conversely, KIF2C high expression is associated with good DFS (HR = 0.53, COX *p* = 0.013566) in colorectal cancer (Figure 2L). For blood cancer (Figures 2M,N), *KIF2C* presented the opposite manner in OS (HR = 0.53, COX *p* = 0.017) and DSS (HR = 2.53, COX *p* = 0.000363). For other cancers, no statistically significant associations were observed.

**FIGURE 3:**
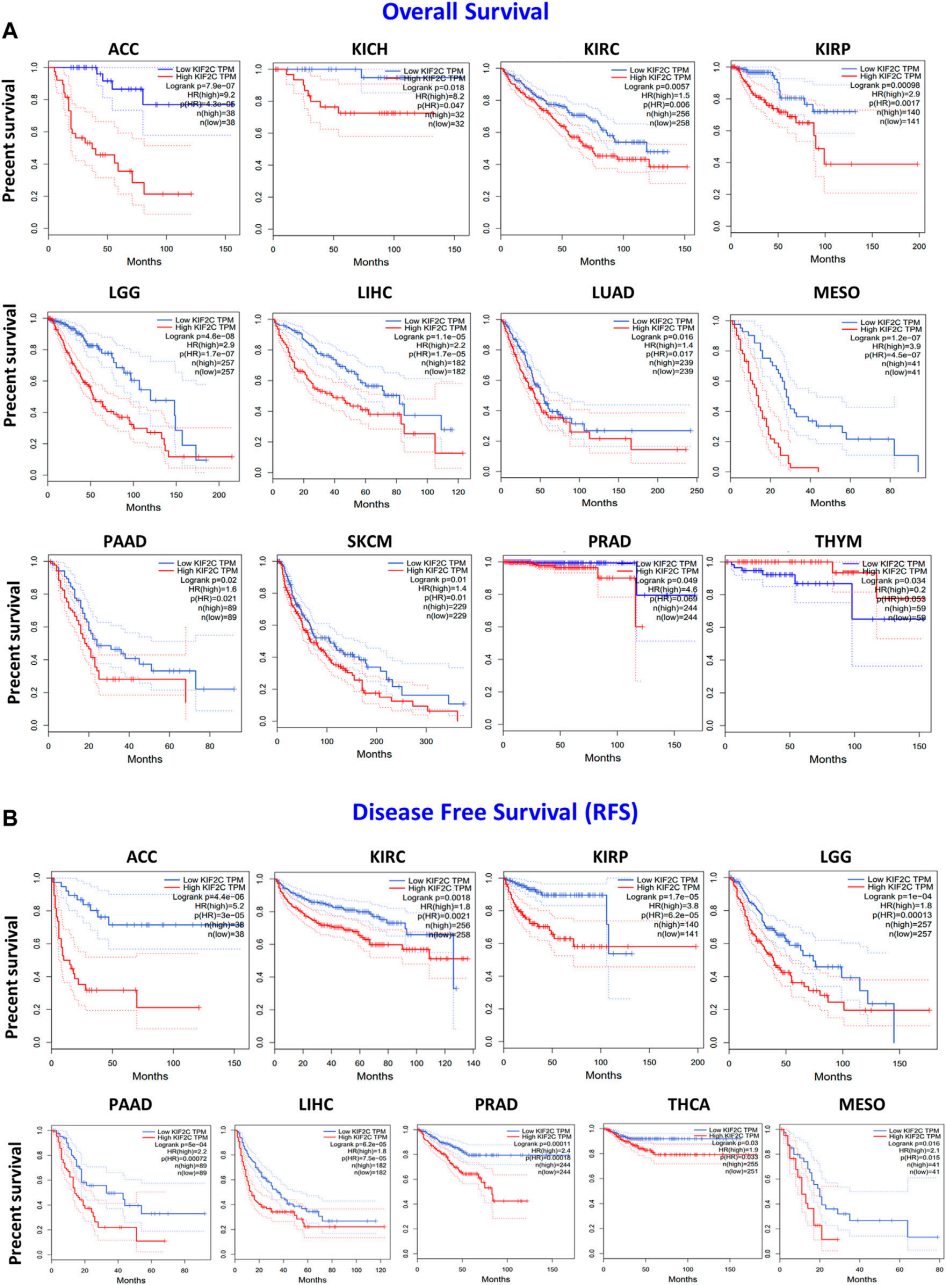
*KIF2C* expression and its association with survival in cancers based on TCGA data analysis. GEPIA2 software was used to analyze **(A)** overall survival rate and **(B)** disease-free survival rate of different tumors in TCGA. Survival plots and Kaplan–Meier curves showing positive results.

To further explore the prognostic value of *KIF2C*, we performed the survival analysis using Kaplan–Meier curves from the GEPIA2. As shown in Figure 3A, higher *KIF2C* expression was associated with poor OS in ACC (*p* < 0.001), KICH (*p* = 0.018), KIRC (*p* = 0.006), KIRP (*p* = 0.0017), LGG (*p* < 0.001), LIHC (*p* < 0.001), LUAD (*p* = 0.016), MESO (*p* < 0.001), PAAD (*p* = 0.02), SKCM (*p* = 0.01), and PRAD (*p* = 0.049), whereas higher KIF2C expression was linked to better prognosis in THYM (*p* = 0.034). As for DFS (Figure 3B), high KIF2C expression was significantly associated with worse DFS in ACC (*p* < 0.001), KIRC (*p* = 0.0018), KIRP (*p* < 0.001), LGG (*p* < 0.001), PAAD (*p* < 0.001), LIHC (*p* < 0.001), PRAD (*p* = 0.00011), THCA (*p* = 0.03), and MESO (*p* = 0.016). These results suggested that higher KIF2C was associated with worse prognosis.

### Analysis of Genetic Alteration for KIF2C in Various Cancers

**FIGURE 4:**
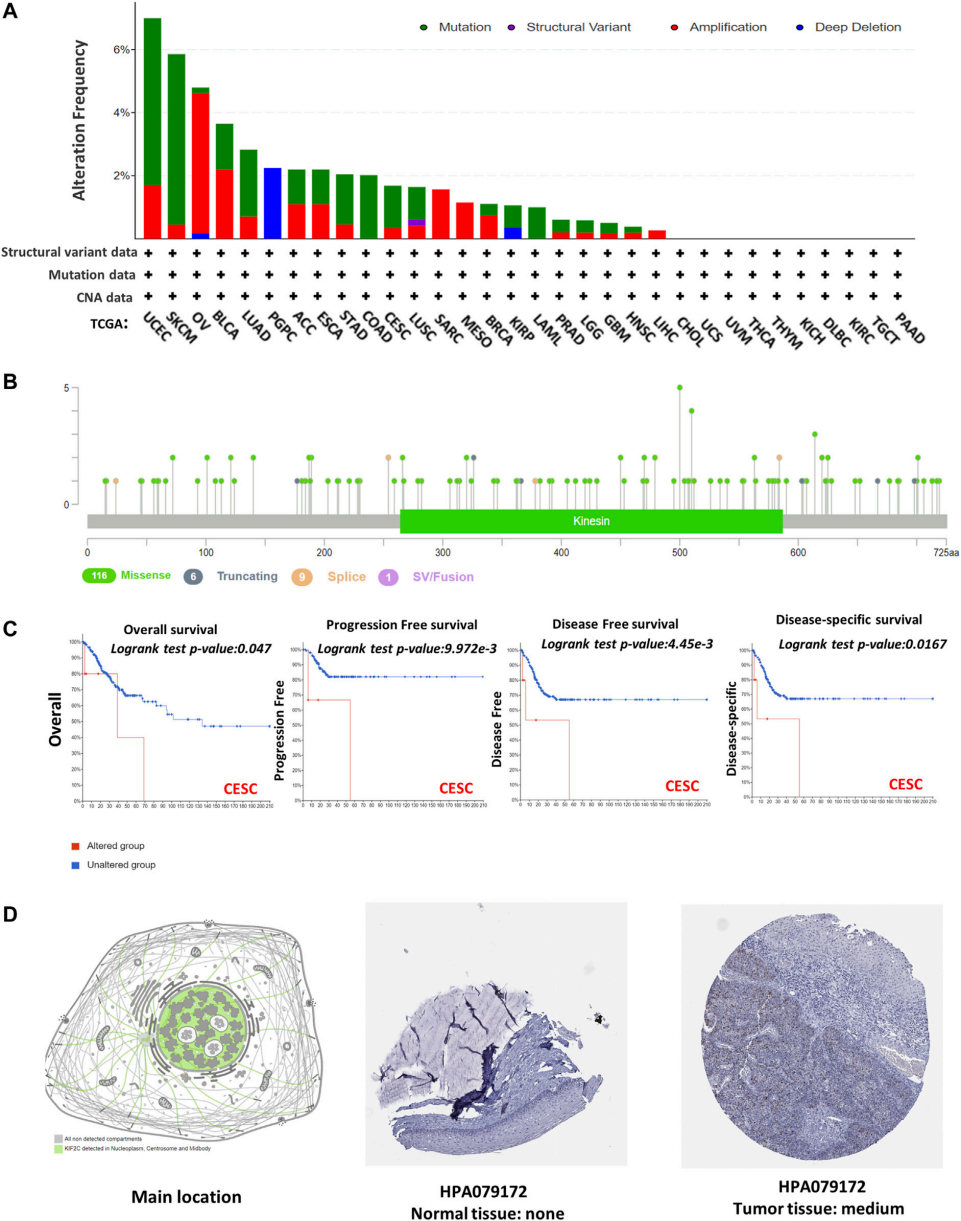
Mutation feature and tissue protein expression of KIF2C in different tumors of TCGA. Mutation features data were obtained using cBioPortal. Alteration frequencies with **(A)** mutation type and **(B)** mutation site are shown. **(C)** Using cBioPortal tool, we analyzed potential correlation between mutation status and overall, disease-specific, disease-free, and progression-free survivals of CESC. Nuclear localization of KIF2C and immunohistochemical results in CESC are shown in **(D).**

*KIF2C* mutations and copy number alterations were investigated using the cBioPortal database. The genetic alteration includes mutation, amplification, deep deletion, and multiple alterations. Notably, the highest mutation frequency (>6%) was observed in UCEC (Figure 4A). The amplification type of CNA was the major type in the OV, which showed an alteration frequency of ∼4% (Figure 4A). The predominant mutation type in PGPC was a deep deletion that resulted in a frameshift (Figure 4A). All COAD and LAML cases with genetic alteration had a mutation of *KIF2C* (Figure 4A). In addition, the amplification of *KIF2C* was present in all SARC, MESO, and LIHC cases (Figure 4A). The information of *KIF2C* genetic alteration is presented in Figure 4B. The missense mutation was the only type of genetic alteration of *KIF2C*. In addition, we also explored the potential association between *KIF2C* genetic alteration and clinical survival rate in various cancers. The CESC patients with KIF2C genetic alteration had poor prognosis in OS (Figure 4C, *p* = 0.047), progression-free survival (Figure 4C, *p* < 0.001), DFS (Figure 4C, *p* < 0.001), and DSS (Figure 4C, *p* = 0.0167). We also used the HPA to investigate the intracellular location and tissue protein expression levels of KIF2C. We found that KIF2C was mostly localized in both cellular nucleus and cytosol (Figure 4D), and it was only detected in CESC tissues (Figure 4D). These results suggested that *KIF2C* could be a potential therapeutic target in CESC.

### KIF2C Was Highly Expressed in CESC Tissue Sample

**FIGURE 5:**
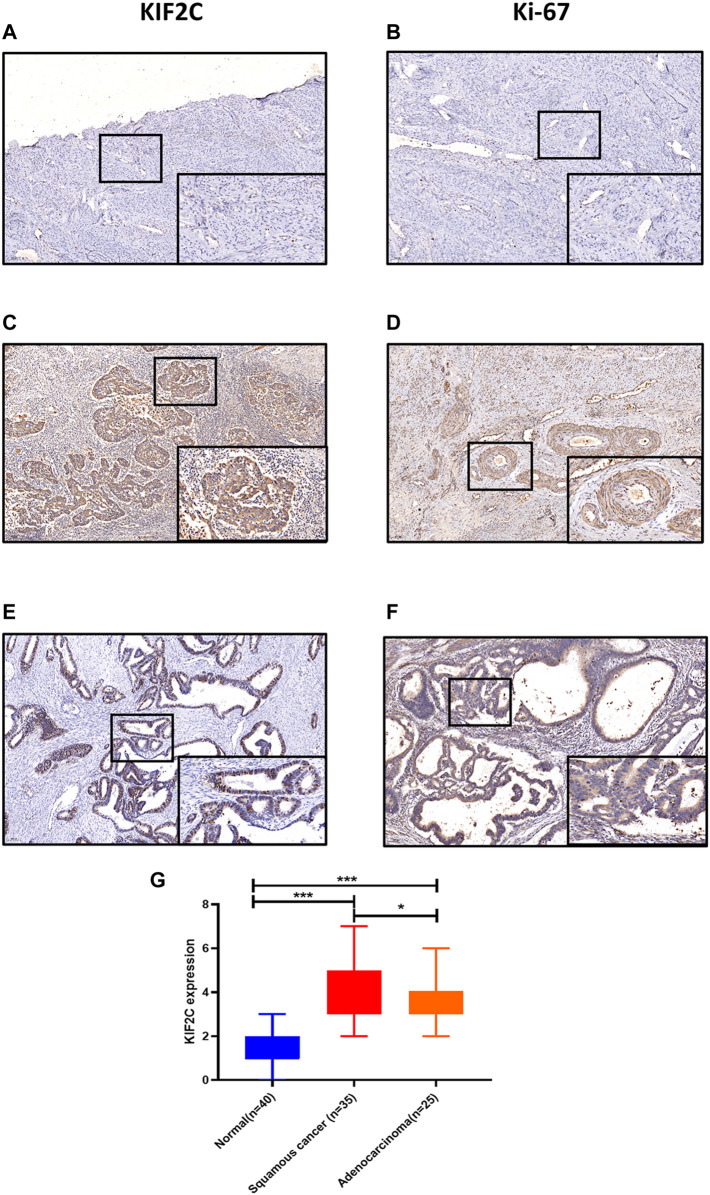
KIF2C was highly expressed in cervical cancer tissues. **(A, B)** Immunohistochemical staining of KIF2C and Ki-67 in normal cervical tissues. **(C, D)** Immunohistochemical staining of KIF2C and Ki-67 in cervical squamous cell carcinoma tissues. **(E,F)** Immunohistochemical staining of KIF2C and Ki-67 in cervical adenocarcinoma tissues. (Insets) Regions with higher magnification. **(G)** Immunohistochemical intensity scores of KIF2C staining in 100 specimens. *p < 0.05, **p < 0.01; ***p < 0.001.

To explore the possibility of KIF2C as a therapeutic target of cervical cancer, we first detected its expression in human tissues. Findings showed that KIF2C was upregulated in cervical cancer tissues compared with normal tissues (Figures 5A,C,E). Not only that, the expression of KIF2C was higher in squamous cervical carcinoma than cervical adenocarcinoma. All three differences were statistically significant (*p* < 0.05, Figure 5G). Besides, we also found that the expression of KIF2C has a similar expression pattern with Ki-67 in cervical tissues (Figures 5B,D,F). This suggested that the expression of KIF2C may be related to the active proliferation of cancer cells.

### Correlation Between *KIF2C* and Clinicopathological Features in Cervical Cancer

To investigate the clinical relevance of KIF2C in cervical cancer, 304 cervical cancer cases from TCGA were analyzed. The results showed that KIF2C expression was correlated with the type of cervical cancer (*p* < 0.05), whereas it was not directly correlated with age, tumor–nodes–metastases stage, 2018 International Federation of Gynecology and Obstetrics stage, lymphatic metastasis, and distant metastasis (Table 2).

**TABLE 2:** Relationship between KIF2C and clinicopathological characteristics in cervical cancer patients.

Infuence factors	Cases	KIF2C		*p* value
		Positive	Negative	
Age (years)				0.305
<40	84	38	46	
≥40	220	114	106	
TNM stage				0.603
I-II	211	108	103	
III-IV	30	14	16	
FIGO stage				0.182
I-II	231	108	123	
III-IV	66	37	29	
Lymphatic metastasis				0.252
Yes	60	36	24	
No	133	68	65	
Distant metastasis				0.126
Yes	10	3	7	
No	116	64	52	
Cancer type				0.006
Adenocarcinoma	52	17	35	
Squamous cell carcinoma	252	135	117	

Chi-square test was used for statistical analysis.

FIGO, 2018 International Federation of Gynecology and Obstetrics; TNM, tumor–nodes–metastases

### Establishment of *KIF2C* Knockdown or Overexpression Cells Using Cervical Cancer Cell Lines

**FIGURE 6:**
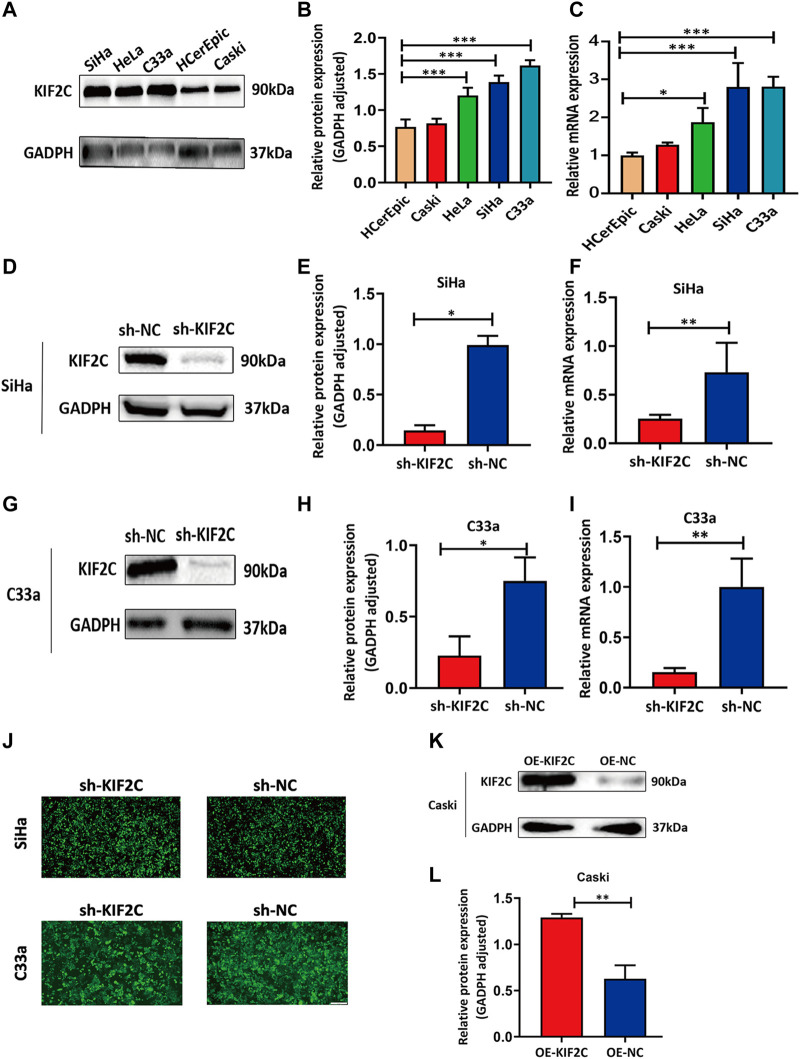
Establishment of *KIF2C* knockdown and overexpression cell lines using cervical cancer cell lines. **(A)** Gray value of KIF2C protein band in SiHa, HeLa, Caski, C33a and HCerEpic; **(B)** relative protein expression of KIF2C in SiHa, HeLa, Caski, C33a, and HCerEpic; **(C)** relative mRNA expression of *KIF2C* in SiHa, HeLa, Caski, C33a, and HCerEpic; **(D)** gray value of KIF2C protein band in SiHa after transfection; **(E)** relative protein expression of KIF2C in SiHa after transfection; **(F)** relative mRNA expression of *KIF2C* in SiHa after transfection; gray value, protein and relative mRNA expression of *KIF2C* in C33a after transfection are shown in Figures 5G–I; **(J)** green fluorescence indicated a transfection efficiency greater than 80%; **(K)** gray value of KIF2C protein band in Caski after transfecting *KIF2C* overexpressed plasmid; **(L)** relative protein expression of *KIF2C* in Caski after transfecting overexpressed plasmid. *p < 0.05; **p < 0.01; ***p < 0.001.

Because we found that *KIF2C* was clinically relevant in cervical cancer in the discussion earlier, we examined the KIF2C expression in various cervical cancer and normal cell lines, including C33a, HeLa, SiHa, Caski, and HCerEpic cell lines. We analyzed mRNA and protein levels of KIF2C using qRT-PCR and Western blot assay in those cells. The results showed that the mRNA or protein levels of KIF2C in C33a, HeLa, and SiHa cells were higher than those in HCerEpic cells (Figures 6A–C). The two cell lines (SiHa and C33a) with the highest KIF2C expression and Caski cell, which had a relatively low level of KIF2C expression, were used for subsequent experiments. To further investigate the role of KIF2C in cervical cancer, we established the stable KIF2C knockdown cell lines using the short hairpin RNA lentiviral system. We confirmed the knockdown efficiency using Western blot, qRT-PCR (Figures 6D–I), and green fluorescent protein (Figure 6J) in SiHa and C33a cells. For overexpression, the Caski cell was transfected with KIF2C plasmid, and the transfection efficiency was verified with Western blot (Figure 6K,L).

### *KIF2C* Knockdown Inhibited the Tumorigenicity of Cervical Cancer Cells

**FIGURE 7:**
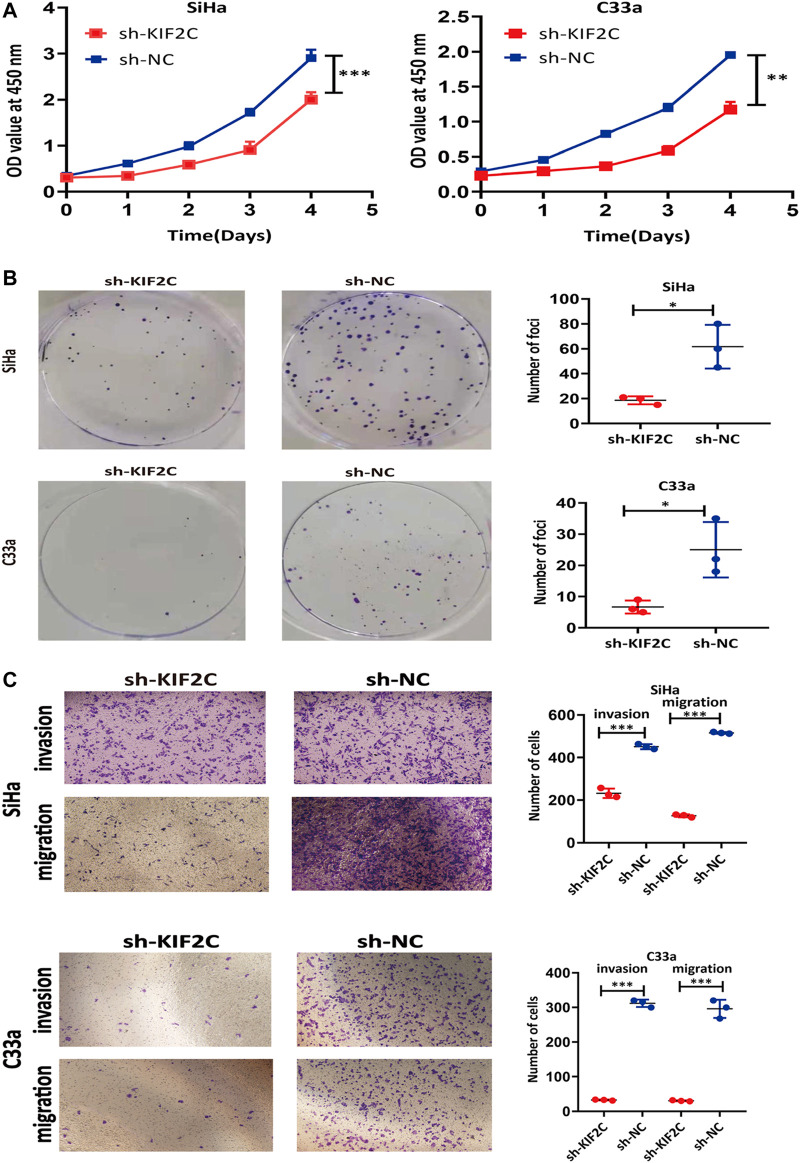
*KIF2C* knockdown inhibited tumorigenicity of cervical cancer cells. CCK-8 assay **(A)** and colony formation assay **(B)** were used to evaluate ability of cell proliferation in cervical cancer cells with *KIF2C* knockdown; dot plots in right showed results of quantitative analyses. **(C)** Migration and invasion of cervical cancer cells were determined after *KIF2C* knockdown and quantification of number of cells in migration and invasion. *p < 0.05; **p < 0.01; ***p < 0.001.

To explore the role of *KIF2C* in cervical cancer cells, we examined the cell viability using CCK-8 assay and clone formation assay in SiHa or C33a cells. The viability of sh-KIF2C SiHa or C33a cells was significantly decreased compared with that of control cells (Figure 7A). These results indicated that the *KIF2C* knockdown inhibited the proliferation of cervical cancer cells. Next, we investigated the role of KIF2C on *in vitro* tumorigenicity in SiHa and C33a cells. The clonal colonies of sh-KIF2C SiHa and C33a cells were also significantly decreased compared with those of control cells (Figure 7B). We then examined whether *KIF2C* knockdown could affect the migration and invasion in SiHa and C33a cells. Results showed that the numbers of migrated or invaded cells in *KIF2C* knockdown groups were significantly lower than those in control groups (Figure 7C). These results indicated that *KIF2C* knockdown suppresses the migration and invasion of cervical cancer cells.

### *KIF2C* Overexpression Promoted the Tumorigenicity of Cervical Cancer Cells

**FIGURE 8:**
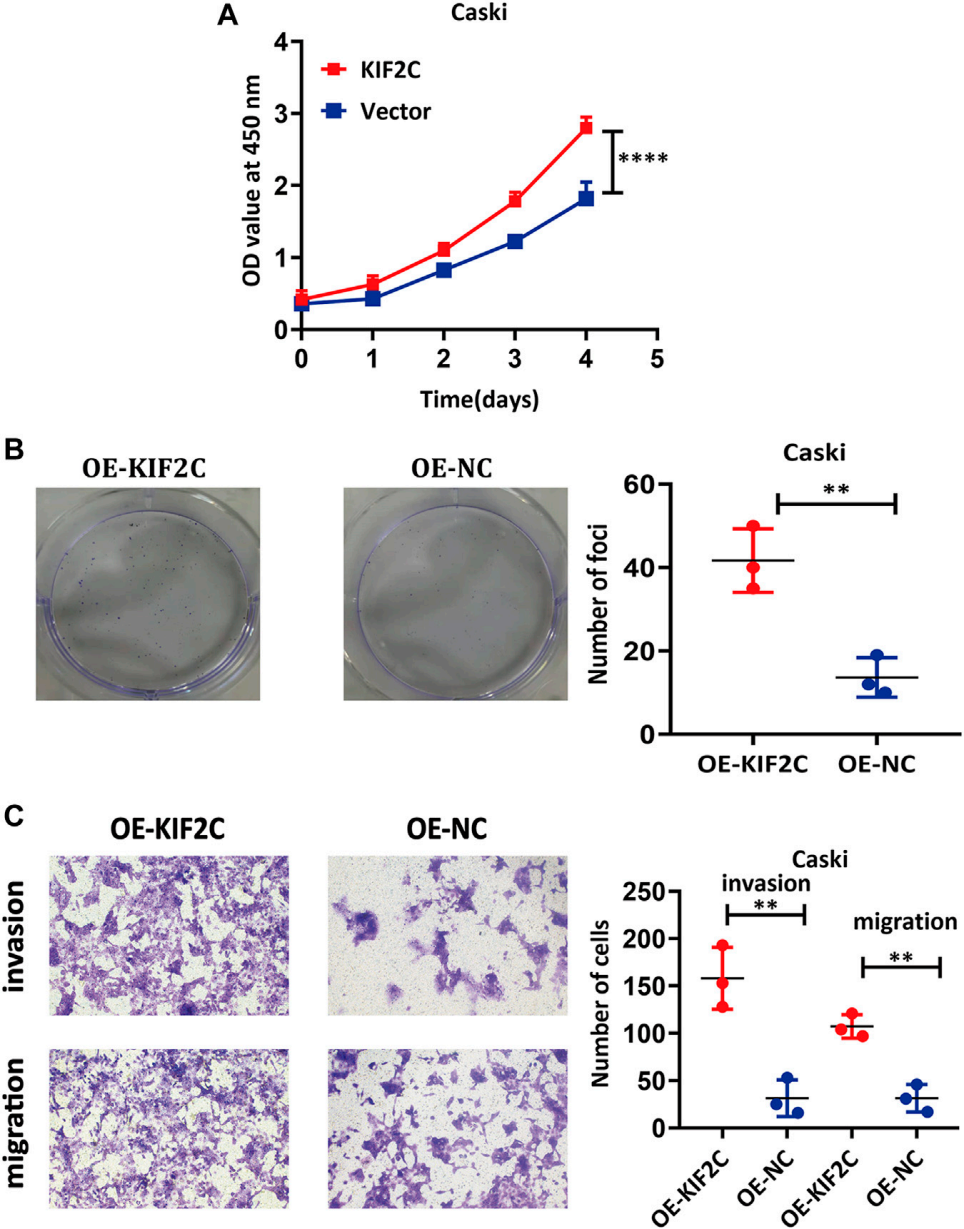
*KIF2C* overexpression promoted tumorigenicity of cervical cancer cells. CCK-8 assay **(A)** and colony formation assay **(B)** were used to evaluate ability of cell proliferation in cervical cancer cells with *KIF2C* overexpression; dot plots in right showed results of quantitative analyses. **(C)** Migration and invasion of cervical cancer cells were determined after *KIF2C* overexpression and quantification of number of cells in migration and invasion. **p < 0.01; ***p < 0.001.

To further probe the role of *KIF2C* in cervical cancer cells, we transfected the *KIF2C* overexpression plasmid into the Caski cell line. CCK-8 assay and clone formation experiment were used to detect the cell viability and clone formation ability. For the CCK-8 assay, the results showed the cell viability was significantly increased in the *KIF2C* overexpression groups compared with the control groups (Figure 8A). For the clone formation experiment, the clonogenic ability of Caski cells, which overexpressed *KIF2C*, was significantly increased (Figure 8B). All findings indicated that *KIF2C* overexpression could promote cell proliferation. By the same token, a transwell assay was performed to examine cell migration and invasion ability, and the results demonstrated that *KIF2C* overexpression can remarkably improve the migration and invasion ability of cervical cancer cells (Figure 8C).

### *KIF2C* Knockdown Regulated Differential Gene Expression

**FIGURE 9:**
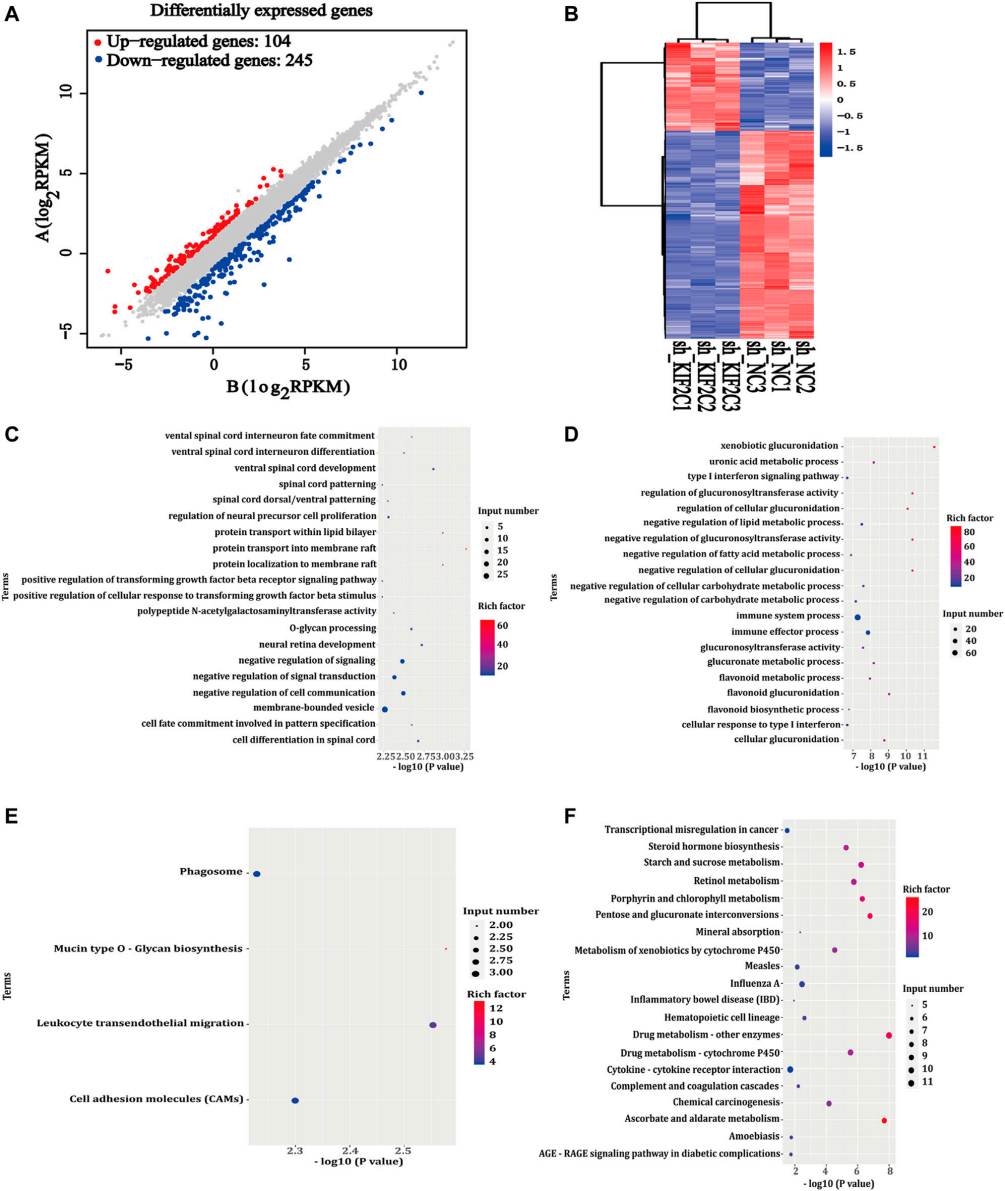
Differentially expressed genes of *KIF2C*. **(A)** Scatter plot of differentially expressed genes between comparison groups; gray dots represent not differentially expressed genes, blue dots represent differentially downregulated genes, and red dots represent differentially upregulated genes; **(B)** leftmost image is overall hierarchical clustering diagram of all differentially expressed genes in all comparison groups, clustered by reads per kilobase of transcript per million reads mapped value, red represents high-expressed genes, and blue represents low-expressed genes. x-axis represents different samples, and y-axis represents gene names; **(C,D)** GO enrichment map of upregulated/downregulated genes; **(E,F)** KEGG enrichment map of upregulated/downregulated genes.

To gain a deeper insight into the mechanism of *KIF2C* expression, we used RNA-seq analysis. We screened the differentially expressed genes using sh-KIF2C SiHa and sh-NC SiHa. Under the screening criteria, which were false discovery rate < 0.05 and |log2FC| >1.5, a total of 349 differentially expressed genes (104 upregulated and 245 downregulated genes) were analyzed (Figure 9A). The results were presented as a heat map, in which blue was the downregulated genes and red was the upregulated genes (Figure 9B). Then, we subjected the 349 genes to GO and KEGG analyses. The results of the GO analysis showed the main pathways in which the upregulated genes were involved in protein transport into membrane raft, protein transport within the lipid bilayer, negative regulation of signal transduction, negative regulation of signaling, etc*.* (Figure 9C). In addition, it also showed the main pathways in which the downregulated genes were involved in xenobiotic glucuronidation, negative regulation of cellular glucuronidation, regulation of glucuronosyltransferase activity, immune system process, and other signaling pathways (Figure 9D). The results of the KEGG analysis revealed that the upregulated genes were mainly enriched in mucin-type O-glycan biosynthesis, leukocyte transendothelial migration, cell adhesion molecules (CAMs), phagosome, etc*.* (Figure 9E), whereas the downregulated genes were mainly enriched in drug metabolism-other enzymes, ascorbate and aldarate metabolism, pentose and glucuronate interconversions, and other related pathways (Figure 9F).

### *KIF2C* Promoted Cervical Cancer Progression Through the p53 Signaling Pathway

**FIGURE 10:**
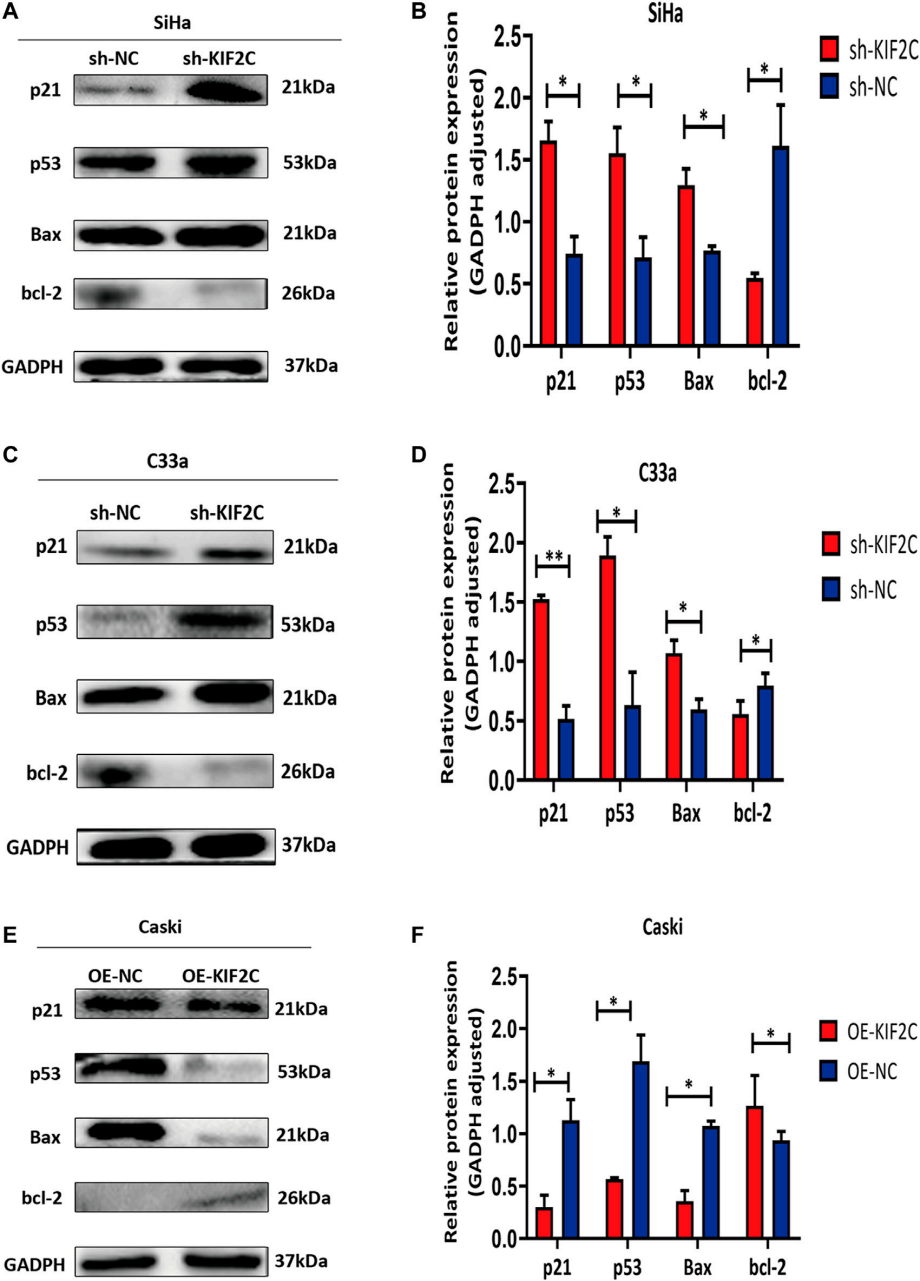
*KIF2C* knockdown promoted activation of p53 signaling pathway. Relative protein expression levels of p21, p53, and Bax were significantly increased, whereas those of bcl-2 was significantly decreased in SiHa **(A,B)** and C33a **(C,D)** cells with KIF2 knockdown. However, expression of proteins mentioned earlier exhibited an opposite trend in presence of KIF2C overexpression **(E,F)**. *p < 0.05, **p < 0.01.

To further experimentally validate the specific mechanism by which *KIF2C* regulates cervical carcinogenesis, we deeply analyzed the KEGG pathway and found that its differentially expressed genes were enriched in the p53 signaling pathway (Supplementary Table S1). There have been some studies showing that alteration of the p53 signaling pathway is involved in the development of cervical cancer. Given that, we further analyzed the expression of proteins involved in the p53 signaling pathways, including p21, p53, bcl-2, and bax. In SiHa and C33a cells with KIF2C knockdown, the expression of p21, p53, and bax proteins was significantly increased, whereas the expression of bcl-2 protein significantly decreased (Figures 10A–D). However, the expression of the proteins mentioned earlier exhibited an opposite trend in the presence of the overexpressed KIF2C (Figures 10E,F). These results indicated that KIF2C might promote cervical cancer progression through the p53 signaling pathway.

### p53 Knockdown Rescued the Inhibitory Effects of *KIF2C* Knockdown in Cervical Cancer

**FIGURE 11:**
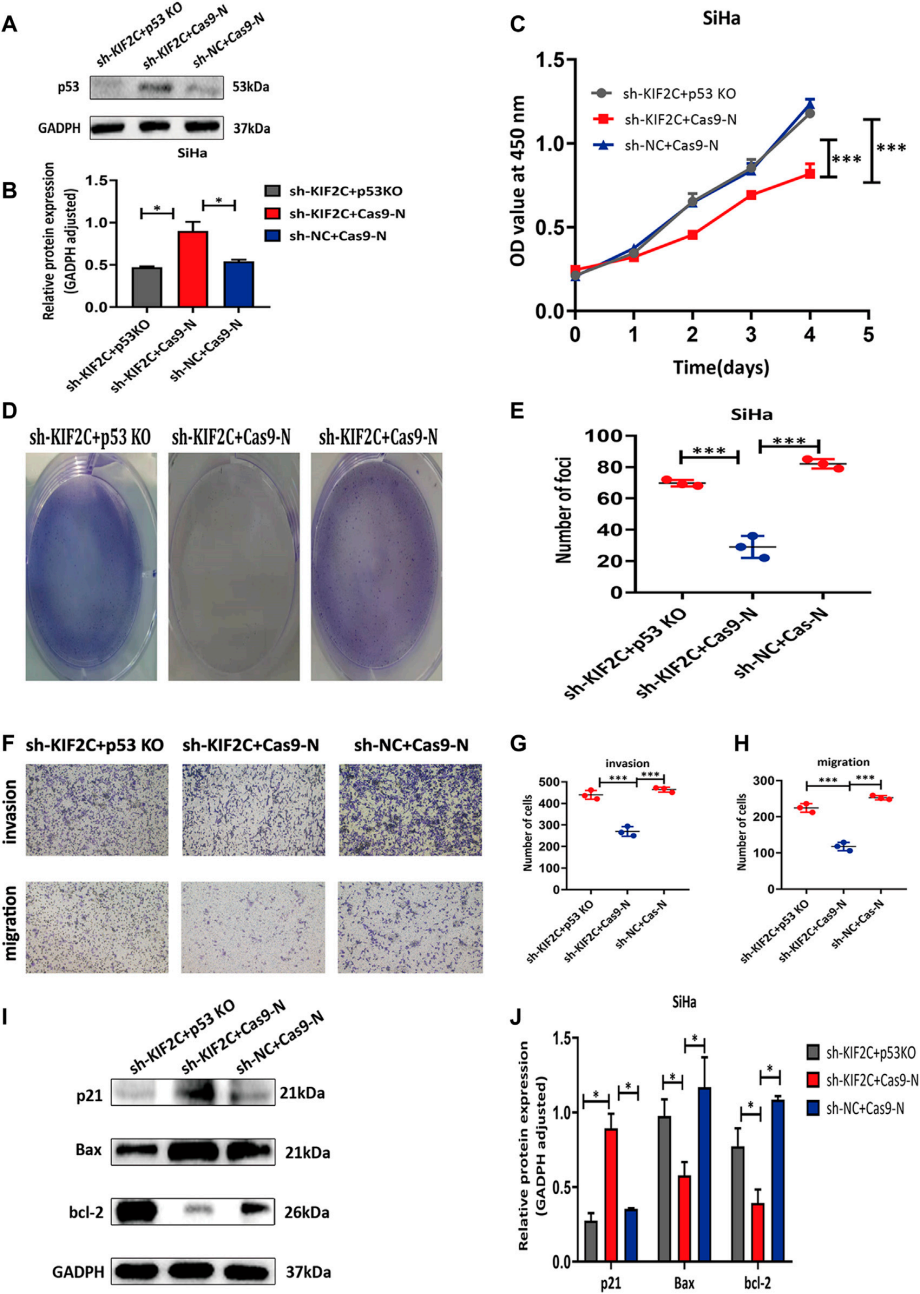
p53 knockdown rescued inhibitory effects of KIF2C in cervical cancer. **(A)** Gray value of p53 protein band in SiHa after transfection; **(B)** relative protein expression of p53 in SiHa after transfection; knockdown of p53 partially reversed KIF2C induced inhibition of proliferation in SiHa cells determined by CCK-8 assay **(C)** and colony formation assay **(D,E)**; knockdown of p53 partially reversed KIF2C induced suppression of invasion and migration **(F)**; bar graphs in **(G,H)** show results of quantitative analyses. **(I)** Gray value of p21, Bax, and bcl-2 protein band in SiHa after transfection; **(J)** relative protein expression of p21, bax, and bcl-2 in SiHa after transfection. *p < 0.05; **p < 0.01; ***p < 0.001.

For additionally exploring the associations of *KIF2C* with p53 signaling pathways, the p53 CRISPR/Cas9 knockout plasmid and control CRISPR/Cas9 plasmid were transfected into the SiHa cell with *KIF2C* stable knockdown. Transfection efficiency was verified using Western blot assay (Figures 11A,B). The outcome revealed that p53 knockdown reversed the prohibiting effect of *KIF2C*-silenced on cell proliferation (Figures 11C–E), migration, and invasion (Figures 11F–H). Besides, we also found that the tendency of p21 and Bax showed the same trend with p53; the level of bcl-2 showed the opposite results (Figures 11I,J). All these data detailed that *KIF2C* might induce cervical cancer cells growth, invasion, and migration by regulating the p53 signaling pathway.

### *KIF2C* Knockdown Suppressed Cervical Tumor Growth in Mouse Models

**FIGURE 12:**
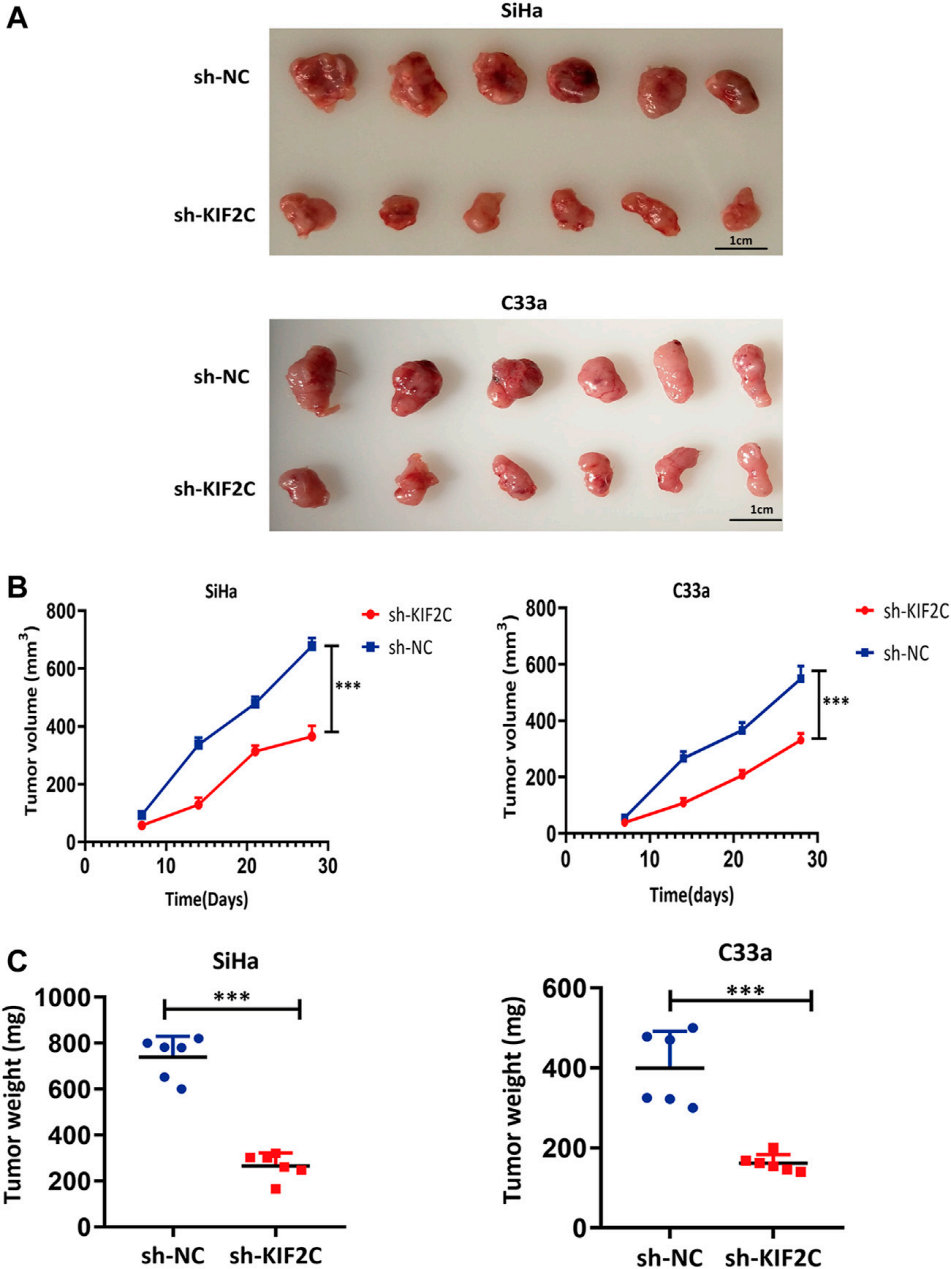
*KIF2C* knockdown suppressed tumor growth *in vivo*. **(A)** Photos of tumor samples from mice; **(B)** curve of tumor volume in mice; **(C)** scatter graph of tumor weight. ***p < 0.001.

To assess the role of *KIF2C* in mouse xenograft models, we subcutaneously inoculated SiHa and C33a cells in the flanks of nude mice. We continuously monitored the tumor growth and measured the volume and weight of tumors for 4 weeks. We found that tumor growth of the sh-KIF2C group was significantly decreased compared with that of the control group (Figure 12A). In addition, the volume and weight of tumors of the sh-KIF2C group were lower than those of the control group (Figures 12B,C). These results indicated that *KIF2C* knockdown played a tumor-suppressive role *in vivo*.

## Discussion

*KIF2C* belongs to the kinesin superfamily proteins, which participate in microtubule depolymerization, microfilament binding to centromere sites, and chromosome segregation, thus regulating the mitotic process of cells (Ritter et al., 2015). Based on its biological function, *KIF2C* has been suggested to have tumor-promoting properties. Previous studies have reported that *KIF2C* is overexpressed in a variety of cancer types. Shimo et al. (2008) used complementary DNA microarray coupled with laser microbeam microdissection gene chip and laser microbeam technology to detect the genomic expression of 81 breast cancers, from which they found that *KIF2C* is a type of *trans*-activated gene. Their further research also showed that KIF2C is highly expressed in breast cancer tissues and other cell lines. Through bioinformatics analysis, Li et al. (2020b) revealed that *KIF2C* is highly expressed in breast cancer and is involved in the worst OS, RFS, and distant metastasis-free survival of breast cancer. In addition to breast cancer, *KIF2C* is also found to be highly expressed in gastric cancer. Nakamura et al. (2007) reported that the expression level of *KIF2C* in gastric cancer tissues is significantly higher than that in adjacent tissues and is significantly correlated with lymph node invasion, lymph node metastasis, and poor prognosis. Moreover, gastric cancer cell lines transfected with the *KIF2C* gene were verified to have a higher proliferation rate and migration capabilities *in vitro*.

In this study, we performed a series of bioinformatics analyses to identify *KIF2C* gene features in pan-cancer. We analyzed the expression characteristics of *KIF2C* according to the TIMER dataset, TCGA dataset, and GEPIA2 dataset. We found that *KIF2C* was frequently abnormally expressed in various types of cancer. *KIF2C* expression was upregulated in many tumor tissues (BLCA, BRCA, CESC, CHOL, COAD, DLBC, ESCA, GBM, HNSC, KIRC, KIRP, LIHC, LUAD, LUSC, OV, PAAD, PCPG, PRAD, READ, SARC, SKCM, STAD, THYM, UCEC, and UCS) compared with the corresponding normal tissues. However, *KIF2C* expression was downregulated in tumor tissues (e.g., TCGT, LAML). Thus, the difference in *KIF2C* expression in various cancers suggested that it might have different biological functions in different types of cancers. Moreover, high expression levels of *KIF2C* were associated with the poor prognosis of most cancers, which strongly suggested that *KIF2C* was a potential prognostic biomarker for tumor treatment. Tumorigenesis is a complex phenomenon, which is often caused by gene mutations (Fan et al., 2021). Based on this, we then studied the characteristics of *KIF2C* gene mutation. The results demonstrated that the main mutation type of *KIF2C* was missense mutation, which was also the most common oncogenic mutation type (Brown et al., 2014). This may indicate that *KIF2C* was closely related to the development of tumors to some extent. Interestingly, we found that the mutation of *KIF2C* was closely related to the survival rate of CESC, which suggested that *KIF2C* might be a potential therapeutic target of CESC. At the same time, immunohistochemical results in public datasets also showed that KIF2C was only highly expressed in cervical cancer tissue. This prompted us to investigate further the biological function of *KIF2C* in cervical cancer, which might provide new druggable targets and novel possibilities for cervical cancer therapy.

To explore these possibilities, we first studied the expression of KIF2C in human tissues. Immunohistochemical staining showed that KIF2C was highly expressed in cervical cancer tissues. More than that, there were some similarities in expression levels between Ki-67 and KIF2C. This suggested a latent relationship for KIF2C with the proliferation of cervical cancer. To further investigate the correlation between KIF2C and clinicopathological characteristics, we performed a statistical analysis using the raw data from TCGA database. We found that the expression of KIF2C was dependent on the tumor types, whereas it had no direct correlation with tumor stage, lymph node invasion, and distant metastasis. Therefore, we assumed that *KIF2C* might be involved in the occurrence and development of cervical carcinoma. KIF2C was highly expressed in most cervical cancer cells. Downregulation of the *KIF2C* expression could significantly inhibit the biological functions of cervical cancer cell lines both *in vitro* and *in vivo*. In contrast, the overexpression of *KIF2C* had the opposite effects. These results also suggest that *KIF2C* may be a potential gene contributing to cervical carcinoma development.

The *p53* gene is a tumor suppressor gene that has the highest correlation with human tumorigenesis among genes that have been discovered so far (Levine, 2020). The gene exerts its growth inhibitory effect by activating and interacting with a variety of signaling pathways (Kasteri et al., 2018). As a key transcription factor, p53 has been found to be inactive at both gene and protein levels in a variety of human cancers. Its upregulation in cancer cells may prevent the proliferation of cancer cells by promoting cell cycle arrest and apoptosis (Wang et al., 2015). For example, *SNRPB* can promote the occurrence and development of cervical cancer by inhibiting the expression of *p53* (Zhu et al., 2020). In addition, in cervical cancer, *ISG15* can upregulate and activate *p53*, which in turn causes the inhibition of proliferation and growth of cancer cells (Zhou et al., 2017). Kong et al. (2015) have reported that *VTRNA2-1-5p* is a direct regulator of *p53*; the inhibition of *VTRNA2-1-5p* can reduce the invasion, proliferation, and tumorigenicity of cervical cancer cells and can increase cell apoptosis and *p53* expression. Furthermore, because *p53* plays a central role in cellular emergency response, deletion or mutation of the *p53* gene can lead to the dysfunctions of upstream regulators or downstream effectors in cancer cells (Miedl et al., 2020). The development of therapy based on the p53 pathway has always faced a huge challenge. Many strategies targeting the p53 pathway have been developed, including the restoration of p53 function, the inhibition of p53-MDM2 interaction, and the conversion of mutant *p53* into wild-type *p53* by gene therapy, the targeting of p53 family proteins, the elimination of mutant *p53*, and the development of p53-based vaccines (Ladds and Laín, 2019). Herein, we found that the downregulation of *KIF2C* causes the activation of the p53 signaling pathway, whereas the upregulation of *KIF2C* inhibits the activation of the p53 signaling pathway. To further confirm the mechanisms by which *KIF2C* regulates cervical cancer formation and development, we applied p53 CRISPR/Cas9 knockout plasmid in Siha cells to rescue the phenotype induced by KIF2C. Indeed, the knockdown of p53 partially reversed the oncogenic effects of KIF2C. Thus, we assumed that *KIF2C* might promote the development of cervical cancer by inhibiting the activation of the p53 signaling pathway. Whether or not this finding can lead to the development of a therapeutic strategy targeting the p53 pathway of cervical cancer, it provides clues and directions for targeted therapy and combined radiotherapy and chemotherapy of cervical cancer, which will be the main topics for our future research.

## Conclusion

In summary, the results of this pan-cancer analysis showed that the abnormal expression of *KIF2C* was associated with poor prognosis of different cancer types. We also found that *KIF2C* promoted cervical cancer cell proliferation by suppressing the p53 signaling pathway. These findings suggest the potential use of *KIF2C* as a therapeutic target for the treatment of cervical cancer.

## Data Availability

The datasets presented in this study can be found in online repositories. The names of the repository/repositories and accession number(s) can be found in the article/Supplementary Material.
