# Supplementary material for: Characterization of Kinesin Family Member 2C as a Proto-Oncogene in Cervical Cancer
Source: Front Pharmacol. 2022 Jan 27;12:785981. doi: 10.3389/fphar.2021.785981 (PMC8828917; doi:10.3389/fphar.2021.785981)
Supplement: Supplementary file 1 [file Table1.DOCX]

Supplementary Material

**Table 1 KEGG pathway of differentially expressed genes**

| **Term** | ***P v*alue** | **Term** | ***P* value** |
| --- | --- | --- | --- |
| Drug metabolism - other enzymes | 8.44E-09 | Complement and coagulation cascades | 0.005542651 |
| Ascorbate and aldarate metabolism | 1.74E-08 | Measles | 0.0062541 |
| Pentose and glucuronate interconversions | 1.41E-07 | Inflammatory bowel disease (IBD) | 0.011151719 |
| Porphyrin and chlorophyll metabolism | 4.36E-07 | Amoebiasis | 0.015565056 |
| Starch and sucrose metabolism | 5.10E-07 | AGE-RAGE signaling pathway in diabetic complications | 0.016234955 |
| Retinol metabolism | 1.49E-06 | Cytokine-cytokine receptor interaction | 0.016701353 |
| Drug metabolism - cytochrome P450 | 2.42E-06 | Transcriptional misregulation in cancer | 0.027260456 |
| Steroid hormone biosynthesis | 4.69E-06 | Herpes simplex infection | 0.032000527 |
| Metabolism of xenobiotics by cytochrome P450 | 2.50E-05 | Prion diseases | 0.034878358 |
| Chemical carcinogenesis | 5.76E-05 | Cytosolic DNA-sensing pathway | 0.040018018 |
| Hematopoietic cell lineage | 0.00220332 | Nitrogen metabolism | 0.049461958 |
| Influenza A | 0.002993157 | p53 signaling pathway | 0.049754384 |
| Mineral absorption | 0.004443646 | Amoebiasis | 0.015565056 |
